# Fingerprint Presentation Attack Detection Utilizing Spatio-Temporal Features

**DOI:** 10.3390/s21062059

**Published:** 2021-03-15

**Authors:** Anas Husseis, Judith Liu-Jimenez, Raul Sanchez-Reillo

**Affiliations:** University Group for ID Technologies (GUTI), University Carlos III of Madrid (UC3M), Av. de la Universidad 30, 28911 Madrid, Spain; jliu@ing.uc3m.es (J.L.-J.); rsreillo@ing.uc3m.es (R.S.-R.)

**Keywords:** fingerprint, presentation attack, presentation attack detection, anti-spoofing

## Abstract

This paper presents a novel mechanism for fingerprint dynamic presentation attack detection. We utilize five spatio-temporal feature extractors to efficiently eliminate and mitigate different presentation attack species. The feature extractors are selected such that the fingerprint ridge/valley pattern is consolidated with the temporal variations within the pattern in fingerprint videos. An SVM classification scheme, with a second degree polynomial kernel, is used in our presentation attack detection subsystem to classify bona fide and attack presentations. The experiment protocol and evaluation are conducted following the ISO/IEC 30107-3:2017 standard. Our proposed approach demonstrates efficient capability of detecting presentation attacks with significantly low BPCER where BPCER is 1.11% for an optical sensor and 3.89% for a thermal sensor at 5% APCER for both.

## 1. Introduction

Fingerprint recognition is one of the oldest and most prevalent biometric modalities. It has shown attractive features such as high accuracy and user convenience; accordingly, it has been applied in applications such as forensics, identity control, physical access control, and mobile devices. A recent study by Juniper anticipates having 4.5 billion mobile devices using fingerprint sensors by 2030 [1].

Unfortunately, the use of a biometric sub-system for authentication processes does not imply that the system is secured. The generic biometric scheme is vulnerable at different points starting from the sensor to the recognition score/decision [2]. Based on those vulnerabilities, biometric security is categorized in two main areas: (a) electronic security which concerns the digital process of the captured biometric trait (b) physical security which questions whether the biometric trait presentation is performed by a bona fide (i.e., genuine user) or by an attacker. This investigation is tended to focus on the second type and propose a potential software countermeasure.

Presentation Attack (PA), informally known as spoofing attack, is defined as a suspicious presentation that aims to manipulate the biometric decision using a Presentation Attack Instrument (PAI). The definition implicitly refers to two classes of attackers (a) concealer: aims to evade being recognized as him/herself (b) impersonator: seeks to claim an identity other than himself. In both cases, the attack might be performed with the bona fide cooperation, e.g., research studies, or without the bona fide consent, e.g., identity theft.

Despite the fact that fingerprint ridge/valley patterns are unique, fingerprints have other phenomena such as perspiration which causes the moisturized skin, consequently, fingerprints leave traces at touched surfaces. By using proper methods and tools, those traces can be captured and used to duplicate a PAI in order to impersonate one’s identity. A group of forensic researchers has conducted an experiment demonstrating that fingerprint traces can be captured from problematic metal surfaces after over 26 days of deposition [3]. The experiment involves a sophisticated method along with advanced tools, which must be considered when calculating the attack potential, but it proves the possibility of capturing latent fingerprints when proper method and tools exist (Table 1).

In order to overcome the issue of PA, researchers have been investigating Presentation Attack Detection (PAD) mechanisms that are capable of eliminating or mitigating PAs. PA and PAD on fingerprint recognition have been widely studied in different investigations [4,13,14,15]. In our previous work [14], we classified PAs considering the attacker’s intention, the used materials for creating the PAI, and whether a PAI contains dynamic or static information. On the other hand, different taxonomies have been proposed to classify PAD mechanisms [15]: (a) hardware/software classification sorts the PAD mechanisms by implying the necessity of modifying the hardware design of the biometric sensor, (b) dynamic/static classification clarify whether the temporal biometric information is needed for a PAD mechanism, and (c) collateral-means/natural-phenomena classification investigate whether the PAD features are natural characteristics of the biometric trait or just collateral information.

A key observation regarding the literature of fingerprint PAD mechanisms is that most studies tend to study the static fingerprint pattern, e.g., 2-D textures and fingerprint quality, rather than fingerprint dynamic features. This can be explained by the fact that collecting dynamic datasets requires extensive time, effort, and expertise which consequently had led to limited dynamic datasets. In addition, integrating dynamic PAD algorithms into the biometric system may require higher computational power and potentially adds more load to the overall system. Section II briefly demonstrates the literature studies about dynamic fingerprint PAD and conducts an accuracy performance comparison between those mechanisms.

Based on these disadvantages, one may ask what the advantages are of analyzing fingerprint dynamics. The primary motivation for this study is that when expert attackers perform attacks, the 2-D impression of the attack presentation resembles the genuine fingerprint pattern, leading to a higher possibility that the attack will be classified as a bona fide presentation. In this context, two recent investigations were carried out to support this claim. First, it has been shown by Goicoechea [16] that attackers with an advanced level of knowledge and expertise can perform attacks with a higher success rate when considering black box fingerprint systems (i.e., mobile devices). Secondly, Casula et al. [17] had conducted an experiment that compares the accuracy of the most efficient state-of-the-art static PAD mechanisms considering: (1) expert attacks called “ScreenSpoof” and (2) LiveDet 2019 attacks. Their results showed that ScreenSpoof attacks decrease the full system accuracy by increasing the Impostor Attack Presentation Match Rate (IAPMR) from 8.7% to 22.8% for the ZJUT mechanism and 14.2% for the JLW mechanism.

In this paper, we propose a PAD mechanism that exploits the dynamic texture of the fingerprint as the discriminative foundation. The dynamic model was chosen because we have experimentally noticed that genuine fingerprint presentations demonstrate a unique development of the ridge/valley pattern due to natural phenomena such as elasticity and perspiration. Moreover, PAs have shown perceptual and statistical dynamic differences as shown in our previous work [5]. Thus, five state-of-the-art dynamic texture descriptors are selected to investigate fingerprint spatiotemporal features aiming to obtain high PAD classification accuracy.

The experimental protocol and evaluation methodology have been conducted following the standard ISO/IEC 30107-3:2017—“Information technology—Biometric presentation attack detection—Part 3: Testing and reporting” [18]. Our proposed PAD subsystem demonstrates the capability of detecting PAs while having a low proportion of misclassified bona fide presentation.

The importance of this work lies in consolidating the spatial fingerprint features of the fingerprint impression with the temporal variations by investigating fingerprint videos instead of studying static fingerprint impressions. This paper investigate three groups of spatio-temporal features: (1) local features extracted from 3-D patches, (2) local features extracted from the XY, XT, and YT planes, and (3) global features extracted from the complete fingerprint video. Our results show an accuracy improvement over the dynamic methods that combine the 2-D features of the fingerprint sequence. Moreover, it is noticed that the first group of features achieves the highest accuracy for the optical technology and the second group performs the best for the thermal technology.

The rest of this paper is structured as follows. Section 2 presents a brief overview of the related work. In the Section 3, we describe the framework of the proposed PAD subsystem. The experiment is characterized in Section 4. Section 5 reports and discusses the experimental results. Finally, we draw our conclusions in Section 6.

## 2. Related Work

In this section, we propose a two-level illustration for the State of the Art (SoA) investigations. We first focus on the SoA in dynamic fingerprint PAD mechanisms and report a performance analysis. The second level concerns the applications of dynamic texture in the biometric systems.

### 2.1. Dynamic Fingerprint Pad Mechanisms

Existing dynamic PAD mechanisms can be categorized into two main classes: perspiration based and ridge distortion based mechanisms. Perspiration based mechanisms rely on the fact that genuine fingerprints naturally produce moisture from the pores, this moisture diffuses during the interaction with the sensor surface resulting in a darker image as time goes by. Ridge distortion mechanisms base on the claim that bona fide and attack presentations produce significantly different distortions under certain presentation circumstances such as pressure [6].

Researches on PAD solutions based on fingerprint distortion started roughly in 2006. Antonelli et al. had performed an initial systematic study on skin distortion and showed that artificial fingerprints produce less distortion than natural fingerprints when the presentation is performed with additional pressure and rotation [6]. The study investigated the optical flow in the sequence of images in order to compare the distortion of attacks and genuine presentations.

Later on, Jia et al. analyzed Skin elasticity assuming that the sequence of genuine fingerprints contains an increasing size of the fingerprint impression and higher intensity value [8]. The investigation was conducted considering one attack specie which is gelatin. The corresponding PAD mechanism extracts and combines the correlation coefficient, image intensity, and standard deviation from the sequence of frames of each fingerprint presentation, and finally uses Fisher linear discriminant analysis for classification.

Additionally, Zhang et al. analyzed the fingerprint deformation and modeled the distortion of genuine fingerprints and attacks using a thin plate spline (TPS) [7]. In their study, the presentation was instructed such that the users applied additional pressure in different directions during the data acquisition. The results confirmed that attack presentations cause less deformation than genuine fingerprints due to the differences in the elasticity between the attack and authentic fingerprint.

On the other hand, fingerprint perspiration was investigated in multiple studies aiming to propose accurate and reliable PAD mechanisms. Initial work in fingerprint perspiration was conducted by Derakhshani et al. aiming to study the influence of perspiration on two successive images acquired with five seconds time difference [9]. By studying the energy and general swing, it was reported that the swing is generally higher in genuine fingerprints than attacks; further, the energy of the first image is found to be significantly high in genuine fingerprints compared with the attacks. Parthasaradhi et al. extended the same study to cover electro-optical and optical sensors, additionally, to address the extreme cases of dry and moisturized fingerprints [10].

Abhyankar and Schuckers proposed later to isolate the changing energy of the perspiration pattern and use the energy distribution of the changing coefficients to classify bona fide presentations from attacks [11]. The study was carried out considering (1) different data acquisition protocol where each fingerprint presentation includes two images separated by two seconds and (2) a larger dataset.

More recently, Plesh et al. proposed acquiring dynamic data with specific sensing technology that acquires different color channels aiming to achieve higher accuracy [12]. The data was acquired such that each presentation includes two images 0.625 s apart. The proposed PAD mechanism extracts five dynamic and two static feature sets. The dynamic features were defined to represent intensity variation, perspiration, displacement, background, and foreground analysis. Finally, the classification is performed using a neural network.

Finally, in our recent work [5], we investigated the dynamic statistics of fingerprints in different sensing technologies. Our study demonstrated that genuine fingerprints and attacks have statistical differences that can be used to classify attack and genuine presentations.

Table 1 conducts a PAD performance analysis for literature studies on both categories and shows the used sensors and attack species.

### 2.2. Dynamic Texture: Applications in Biometrics

Dynamic textures are textures with motion [19]. Ideally, a dynamic texture descriptor consolidates 2-D textures in a scene with temporal variations, meaning that information of space and time are obtained simultaneously. There is a vast amount of literature on dynamic texture recognition with application to biometric recognition and analysis, this section highlights some related works in the domain.

In their seminal paper of 2007 [20], Zhao and Pietikäinen proposed a simple approach to extract dynamic textures using Volume Local Binary Patterns (VLBP) and Local Binary Patterns from Three Orthogonal Planes (LBP-TOP). The method had been proposed with application to facial expression recognition and reported over 95% accuracy. Moreover, a recent study on spontaneous facial micro-expression recognition suggested a deep learning model based on spatial and temporal streams and reported 63.53–74.05% accuracy [21].

In 2018, Zhao et al. had carried out an experiment on the applications of the VLBP in face PAD [22]. The authors had evaluated their PAD mechanism considering printed and replay attacks (video attacks). The PAD mechanism had successfully eliminated all printed attacks with 100% accuracy and mitigated replay attacks with 97.38% accuracy.

Additionally, various dynamic descriptors were suggested to categorize human actions. Solmaz et al. [23] extended the GIST descriptor into GIST 3-D and evaluated the method on different datasets, the authors obtained 92% accuracy for classifying 6 action categories. Further, Rahman and See [24] suggested utilizing the Binarized Statistical Image Feature (BSIF) to extract the dynamic features from 3-D salient patches and reported 93.43% accuracy for classifying low-quality videos.

## 3. Proposed Presentation Attack Detection Subsystem

**Figure 1:**
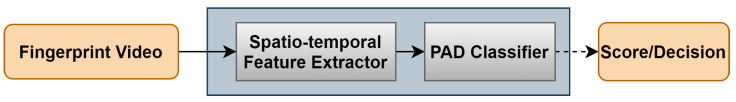
Dynamic PAD subsystem scheme.

The proposed PAD subsystem is designed in a fashion that leverages the dynamic information provided during the fingerprint presentation (Figure 1). Thus, the proposed feature extraction approach suggests exploiting the spatio-temporal features to achieve a robust description that characterizes the complete interaction between the fingerprint and the sensor’s surface. Toward this end, we propose three modes to investigate fingerprint dynamics in frequency and time domains. Five feature extractors are therefore selected to achieve a description that discriminates genuine from attack presentations. By feeding the extracted features into a pre-trained classifier, the PAD subsystem finally decides whether the input video is a bona fide or attack presentation. The following subsections expound the processing modes, feature extractors, and classification method.

### 3.1. Feature Extraction Modes

In order to investigate different aspects of fingerprint dynamics, three feature extraction modes are elaborated in this subsection. The first mode investigates dynamic fingerprint features in the frequency domain whereas a 3-D filter bank is utilized to extract spectral features in a diverse range of scales and orientations. As the video’s frequency components effectively represent the static fingerprint pattern and the temporal variations, it is expected that the differences between natural skin and attack species produce frequency components in different planes. Hence, this mode captures the spatio-temporal information by filtering the video frequency spectrum in different orientations and center frequencies.

The second mode samples the fingerprint video on space-time domain into small 3-D patches, extracts the spatio-temporal features from those samples, and provides the description as the frequency distribution of the extracted features. This mode has two main interesting features, primarily, it has the capacity to define local features in a stack of XY patches so that any anomalous formation in the fingerprint video is detected. Secondly, it provides the possibility of processing the 3-D patches in space-time and/or frequency domains.

The third mode resembles the second mode, a small brick is added after the sampling to decompose the 3-D patches into the Three Orthogonal Planes (TOP) XY, XT, and YT planes. Over the advantages of the second mode, the third mode had proved significantly reduced complexity for the adopted feature extractor while preserving a high accuracy when the local binary patterns are extracted [20].

**Figure 2:**
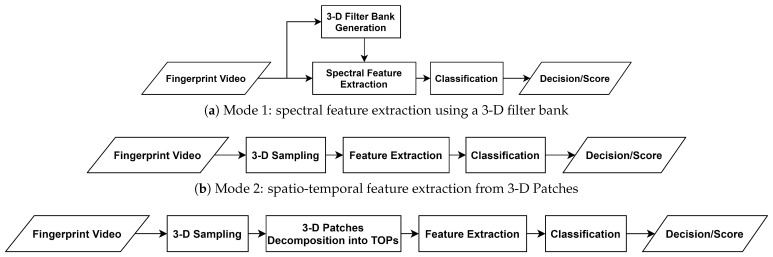
Proposed PAD scheme in different modes.

**Figure 3:**
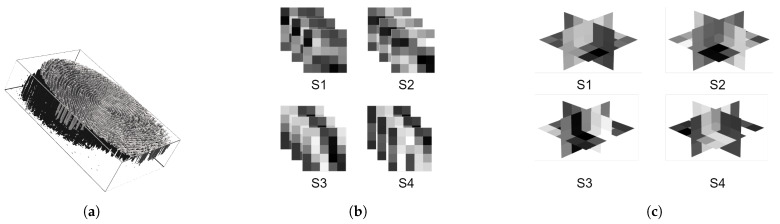
Illustration of 3-D sampling and decomposition. Fingerprint Video (**a**), 3-D patches sized (5×5×3) (**b**), and patches in b decomposed into xy, xt, and yt planes (**c**).

Figure 2 illustrates these modes and Figure 3 shows an example of a fingerprint video and its sampling into 3-D patches and TOPs.

### 3.2. Feature Extractors

The feature extractors were selected in order to comply with the proposed modes, moreover, to analyze the features in spatio-temporal and spectral domains. Table 2 summarizes the proposed scenarios with the corresponding dynamic feature extractors and the following subsections reviews these algorithms.

#### 3.2.1. GIST 3-D Descriptor

GIST 3-D is a global spatio-temporal descriptor that had been proposed for video classification problems. The method integrates the motion information and the scene structure in one feature vector without applying background subtraction or salient point detection at the input video, achieving performance better than SoA dynamic descriptors. In our experiment, the GIST 3-D works as follows: first, the frequency spectrum of the complete fingerprint video is achieved by applying 3-D Discrete Fourier Transform; as computed by Equation (1).

(1) $$\begin{array}{c} F\left(f_{x},f_{y},f_{t}\right)=\frac{1}{MNT}\sum_{x=0}^{M-1}\sum_{y=0}^{N-1}\sum_{t=0}^{T-1}f\left(x,y,t\right)\\ e^{-j2\Pi (\frac{xf_{x}}{M}+\frac{yf_{y}}{N}+\frac{tf_{t}}{T})}, \end{array}$$

Then, a bank of narrow band 3-D Gabor filters G(fr,θ,ϕ) is generated and each 3-D filter Gi(fx,fy,ft) is applied to the frequency spectrum as given by Equation (2). The filter bank is composed by 3-D filters with different orientations and scales, which allows capturing the components at various intervals of the video’s frequency spectrum.

(2) $$\Gamma_{i}\left(f_{x},f_{y},f_{t}\right)=F\left(f_{x},f_{y},f_{t}\right)\left[G_{i}\left(f_{x},f_{y},f_{t}\right)\right],$$

After taking the inverse 3-D DFT as in Equation (3) for each filter in the bank, the output volume is quantized in fixed sub-volumes and the sum of each sub-volume is taken, thus, a feature vector is obtained to represent the video description.

(3) $$\begin{array}{c} H_{i}\left(x,y,t\right)=\sum_{f_{x}=0}^{M-1}\sum_{f_{y}=0}^{N-1}\sum_{f_{t}=0}^{T-1}\Gamma_{i}\left(f_{x},f_{y},f_{t}\right)\\ e^{j2\Pi (\frac{xf_{x}}{M}+\frac{yf_{y}}{N}+\frac{tf_{t}}{T})}, \end{array}$$

#### 3.2.2. Volume Local Binary Patterns

The basic Local Binary Patterns method was extended to VLBP in order to describe the dynamic texture in a sequence of successive images [12]. The algorithm starts by sampling the gray level volume input into small 3D samples considering a certain number of local neighbors (P), time interval (L), and radius (R) in x-y plane, then every neighbor pixel in the 3D sample is given a binary value based on a comparison with the center pixel of the sample. Finally, each binary value is multiplied by a corresponding weight and all results are summed to form the sample’s VLBP_L,P,R_ code; Equation (4). The distribution of the codes is used to compose the dynamic texture feature vector.

(4) $$VLBP_{L,P,R}=\sum_{p=0}^{3P+1}s\left(g_{p}-g_{c}\right)2^{p},$$

where $g_{p}$ and $g_{c}$ correspond to the gray values of the central pixel and neighbours in the 3-D sample.

The authors in [20] also proposed two additional modes for the method: (1) rotation-invariant VLBP mode ($VLBP_{L,P,R}^{ri}$) which is based on the assumption that volume data rotates only around t-axis, (2) uniform VLBP mode ($VLBP_{L,P,R}^{u2}$), where the VLBP histogram consists of uniform patterns (i.e., patterns contain at most 2 bitwise transitions between 0 and 1) and sums up all non-uniform patterns in 1 bin.

#### 3.2.3. Volume Local Phase Quantizer

The VLPQ method [25] is an extension to the local phase quantization which was originally proposed as an image descriptor [26]. VLPQ essentially encodes local Fourier transform’s phase information at low-frequency points. The method consists of three steps: (1) local Fourier transform is applied, using Short Term Fourier Transform (STFT), over M×M×N neighborhood N_x_ centered at each pixel position x using 1-D convolutions for each dimension, (2) the dimensionality of the achieved data is reduced using Principal Component Analysis (PCA), and (3) a scalar quantization is applied to produce an integer value. The histogram of the binary codewords is computed to form the VLPQ_M,N_ feature vector.

#### 3.2.4. Local Binary Patterns from Three Orthogonal Planes

Although VLBP method is interesting, it suffers from two major issues. First, initializing the algorithm with a large number of neighbors P results in a very large number of patterns in the VLBP feature vector, limiting the method’s applicability. Second, choosing a time radius L larger than 1 excludes the frames with a time variance less than L.

To address these issues, VLBP-TOP_L,P,R_ method had been proposed in [20] to concatenate the local binary patterns on the three orthogonal planes: XY-LBP, XT-LBP, and YT-LBP. With this approach, spatial patterns are obtained from XY plane and space-time transitions information is attained from XT and YT planes. As a result, the number of patterns on the feature vector is significantly reduced from $2^{3P+2}$ to $3\times 2^{P}$ which allows considering a large number of neighbors with reduced computational cost, moreover, including neighbor pixels from frames with a time variance less than L, when L is larger than 1.

#### 3.2.5. Local Phase Quantizer from Three Orthogonal Planes

LPQ-TOP_Rx,Ry,Rz_ is implemented by calculating LPQ histograms from three orthogonal planes similar to LBP-TOP. The histograms are normalized and concatenated to form the LPQ-TOP descriptor [25].

### 3.3. PAD Classification

Through our experiment, we have tested different classification algorithms, specifically: Classification Trees, Discriminant Analysis, Naive Bayes, Nearest Neighbors, SVM Classification, and Classification Ensembles. SVM classification has been chosen due to its highest accuracy, while the other classification methods are not considered in this paper. Moreover, we have examined the impact of changing the SVM kernel whereas a second polynomial kernel demonstrated the best accuracy. A binary classification scheme has been utilized to evaluate the PAD subsystem performance and to assess the influence of specific PAI species on system security and usability.

## 4. Experiment

To evaluate the performance of the proposed PAD subsystem, we use the dynamic dataset presented in [5]. In the initial stage of the experiment, a volume segmentation is applied to the database. This sets the input fingerprint videos to the feature extraction step. At this point, we utilize the scheme in Figure 2 to extract the features and train the SVM model. As soon as these steps have been carried out, the testing process is performed, and the PAD subsystem accuracy is assessed.

### 4.1. Database Description

The database had been collected to capture genuine and cooperative-attack presentations as videos using optical and thermal sensors. The database comprises 66 genuine fingerprints (thumb, index middle) taken from both hands of 11 independent subjects, and attacks using seven PAI species. A definite characterization of the protocol applied to produce this database is introduced in [5].

Table 3 summarizes the 3564 bona fide and attack presentations in the database with the corresponding presentation type.

The Common Criteria (CC) defines the attack potential as a function of expertise, resources, and motivation of the attacker. Reporting those aspects in biometric databases is therefore indispensable to the coherence of the PAD evaluation. We thus report that all attacks were carried out by one attacker, he has an advanced knowledge in biometric systems and had proven practical experience in attacking fingerprint sensors embedded in smartphones. Furthermore, the attacker obtained all required materials from local shops and online stores for a very low cost. Accordingly, the attacker has prepared each PAI species with a particular recipe and determined that a PAI can be used multiple times for all species except the Play-Doh instrument where each attack requires a new PAI.

### 4.2. Volume Segmentation

The dataset was collected using optical and thermal sensors where each sensor acquires images with different characteristics. Taking into account the sensors’ features and database characteristics in Table 4, the following subsections illustrate the adopted segmentation techniques.

#### 4.2.1. Thermal Subset

The thermal sensor’s SDK provides a capturing mode that acquires only the central region of the sensor sized 90×128 pixels. Thus, the acquired sequence is already segmented as a stack of 7 frames sized 90×128.

#### 4.2.2. Optical Subset

**Figure 4:**
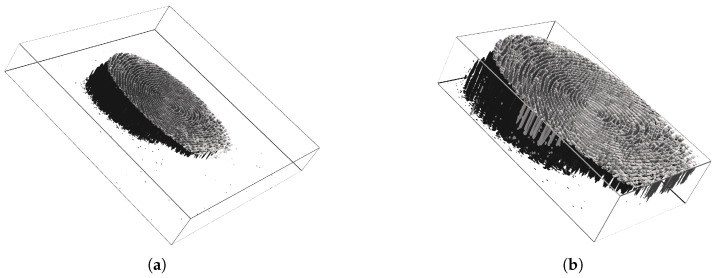
Demonstration of a volume segmentation for a presentation consists of 29 successive frames. Before segmentation sized 375×400 (**a**), and after segmentation 234×145 (**b**). The figures do not reflect the real scale of the fingerprint.

Since our study analyzes the formation of fingerprints, we have implemented a simple volume segmentation tool that creates the boundaries of the entire Interaction between a fingerprint and the sensor and crop the 3-D volume; an example is shown in Figure 4. Then, we have applied the segmentation to the entire subset of the optical sensor before feature extraction.

### 4.3. Experimental Protocol

Each sensor subset is evaluated independently due to the differences in the sensors’ technology, image size, resolution, noise, and capturing rate which produce different video characteristics. For a robust accuracy estimation, we have set a holdout validation scheme where the database is divided into training (55%) and testing (45%) sets. The database division into training/testing is randomized by independent subjects, meaning that presentations of each independent subject is either used for training or testing.

Since the work is focused on the PAD subsystem, we report the error rates following the recommendations of ISO/IEC 30107-3:2017 standard on PAD testing and reporting. The PAD subsystem evaluation determines the system’s capability of detecting attacks taking into account the measurement of false detections.

The following metrics are used in the results to evaluate PAD mechanisms:

- Attack Presentation Classification Error Rate (APCER) presents the proportion of attack presentations incorrectly classified as bona fide presentations. Besides, APCER_PAIS_ is outlined to denote the misclassified attack proportion for a given PAI species;
- Bona Fide Presentation Classification Error Rate (BPCER) presents the proportion of bona fide presentations incorrectly classified as attack presentations;
- Tradeoff Equal Error Rate (TEER) is when APCER and BPCER are equal. We introduce TEER, which is not defined in the standards, to compare with SoA mechanisms that were reported only in terms of TEER, and moreover to prevent the confusion with the conventional EER.

The use of TEER to compare different PAD mechanisms is not recommended because it shows the systems BPCER at different APCER points. It is preferable to evaluate the PAD mechanism in terms of BPCER at fixed APCER, for instance, reporting a PAD mechanism’s BPCER when APCER is 5% is standarized as BPCER20. Furthermore, showing the DET curves [27] provides a precise description of the relationship between APCER and BPCER at different thresholds, allowing better comparison between different mechanisms.

## 5. Results and Discussion

In this section, we assess the accuracy of the proposed PAD scheme and analyze the influence of selecting the feature extractor on the PAD subsystem efficiency.

### 5.1. Impact of PAD Subsystem Mode and Feature Extraction Method

**Figure 5:**
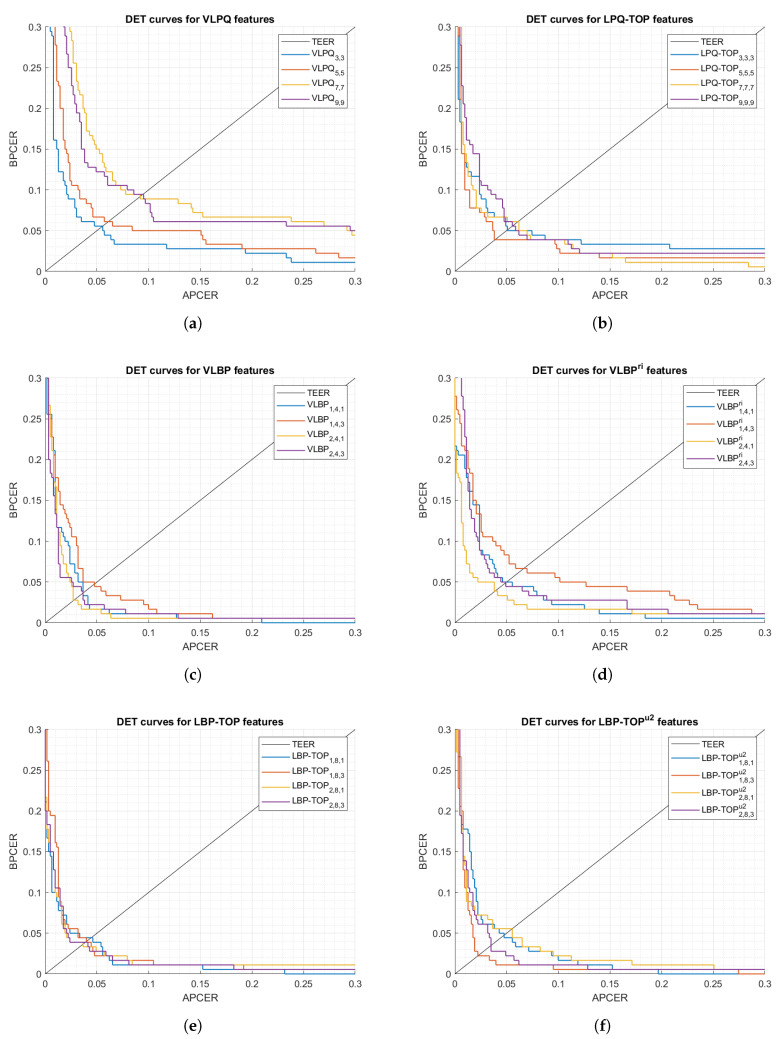
DET curves comparison of the proposed feature extraction algorithms using different parameters (optical sensor).

**Figure 6:**
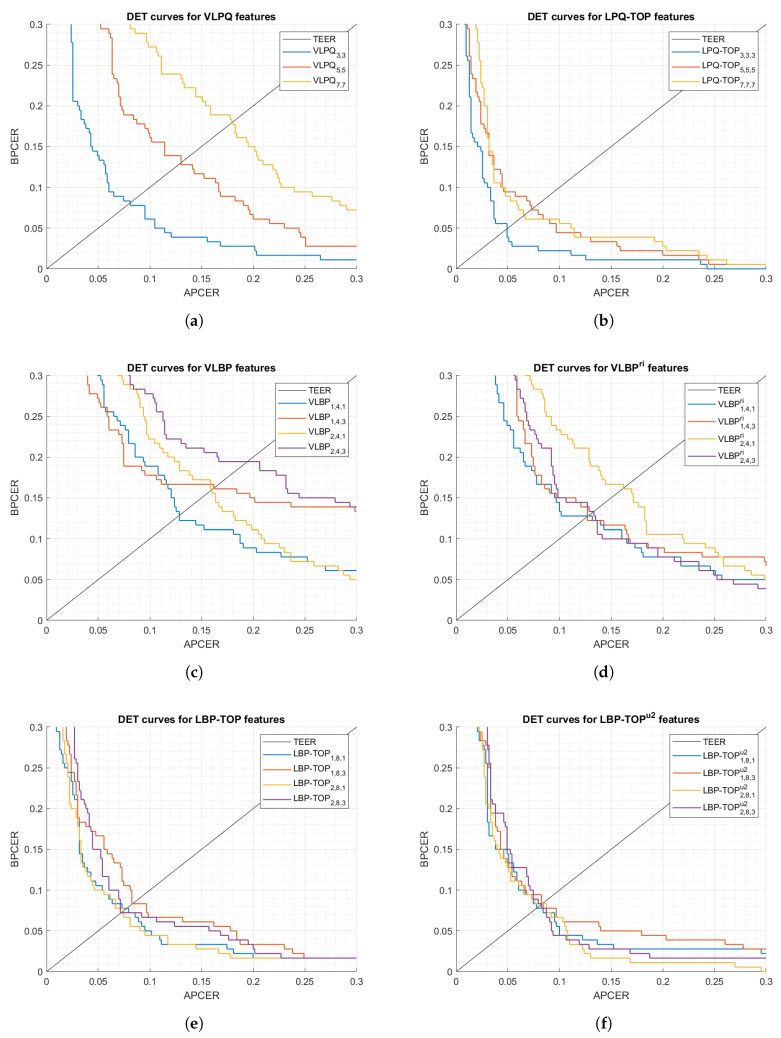
DET curves comparison of the proposed feature extraction algorithms using different parameters (thermal sensor).

The first set of analyses examined the impact of (i) the size of 3-D samples used in the processing mode, and (ii) selecting rotation invariant or uniform features, on the feature extractor performance. Figure 5 and Figure 6 show DET curves for VLBP, LBP-TOP, VLPQ, and LPQ-TOP with the corresponding sampling parameters. The figures confirm that 3-D spectral features (i.e., VLPQ and LPQ-TOP) performs better at smaller sampling size, and the accuracy degrades considerably when comparing the smallest and largest sampling size. An exception is noticed for the LPQ-TOP when executed on the optical sensor. On the other hand, 3-D spatio-temporal features (i.e., VLBP and LBP-TOP) have not revealed a general correlation between sampling size and accuracy. However, it is evident that rotation invariant and uniform features do not necessarily improve the accuracy in most of the cases but nonetheless no significant degradation has taken place after considering those features.

Table 5 and Table 6 detail the results categorized by the feature extraction method. We have selected multiple thresholds: (i) TEER, (ii) APCER = 5%, and (iii) APCER = 2.5% to evaluate the methods at different security levels. The tables reveal the total number of the misclassified bona fide/attack presentations at each threshold. It is worthwhile noting that testing data, which corresponds to 5 independent subjects, consists of 630 attack and 180 bona fide presentations.

**Figure 7:**
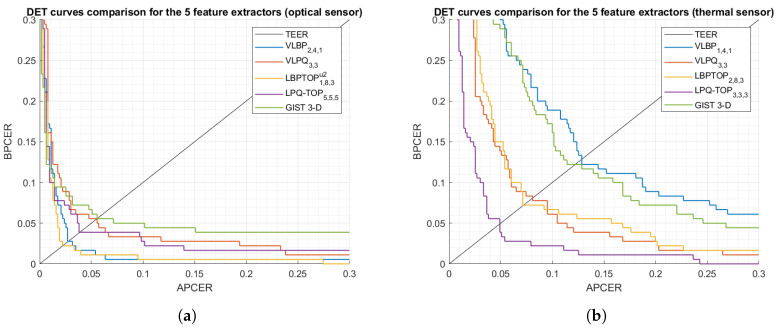
DET curves comparison of the proposed PAD subsystem using five feature extractors.

We then carry out a performance comparison between the five dynamic feature extraction methods (Figure 7) by selecting the methods’ best parameters from Table 5 and Table 6. Note that those parameters had been chosen empirically, thus they might not be optimal for the suggested feature extractors in the context of our experiment.

The most striking result to emerge from Figure 7 is the achievement of significantly low BPCER20, where the system security remains high (low APCER) with low bona fide rejects (low BPCER), that is to say, these results offer powerful evidence for the fact that a genuine fingerprint provides sufficiently discriminative dynamic information that distinguishes it from attacks.

### 5.2. Impact of Sensing Technology

We next investigate the robustness of the proposed PAD subsystem when different fingerprint sensing technologies are used, explicitly, we compare the PAD accuracy for the thermal and optical sensors (Figure 7) in terms of BPCER20. We observe from Table 7 that the accuracy of the PAD subsystem for the optical sensor has an advantage over the thermal sensor. The distinction appears to be well substantiated by the higher frame rate, image size, and resolution in the optical sensor which allows to precisely capture the fingerprint/PAI formation; i.e., spatio-temporal information. Moreover, each presentation in the thermal sensor is captured over roughly 5 s while in the optical sensor, a presentation can be captured in 0.5 s including 10 successive frames.

### 5.3. Impact of Attack Species

This section expounds the results in section A seeking to point out the attack potential for each PAI species. The classification results are shown considering the SVM classification decision in Table 8 and Table 9.

As expected, the tables prove that different attack species have different attack potential considering a target sensor/PAD method. The PAD subsystem has been capable of eliminating some of the attack species and mitigate the rest of the species. Even though the overall performance for the optical sensor has been proven to be higher than the thermal sensor, a comparison between Table 8 and Table 9 demonstrates that the thermal sensor is notably vulnerable to white glue attacks but resistant to the rest of attack species. On the other hand, the optical sensor shows either low or 0% APCER for all attack species.

### 5.4. Accuracy Comparison with SoA Mechanisms

To conduct a comparison between different PAD mechanisms, we emphasize the importance of considering the differences between experimental protocols, used databases, and evaluation methodologies. These factors refer to a certain attack potential to specific database/technology and evaluated using defined metrics.

In the previous sections, these factors and the obtained results have been characterized to a considerable extent in order to allow the reader to compare our proposed PAD mechanism with SoA mechanisms, as shown in Table 10. We note that our results demonstrate an improvement to the SoA dynamic methods for both sensing technologies.

## 6. Summary and Conclusions

In this paper, we present a novel fingerprint PAD approach in the dynamic scenario. We propose three modes to investigate the spatio-temporal and spectral features in fingerprint videos. We utilize five dynamic feature extractors to leverage the fingerprint features in space and time, then a binary SVM is used for classifying bona fide and attack presentations. The PAD mechanism is assessed using a database that was collected using optical and thermal sensors and consists of 792 bona fide presentations taken from 66 genuine fingerprints and 2772 attack presentations performed by an attacker using 7 PAI species.

The significance of the proposed approach is that it integrates the effect of all natural fingerprint phenomena from the acquired video using dynamic descriptors. For instance, the intensity of the fingerprint impression varies through the time series images due to the combination of: the pressure caused by the internal finger bone, the skin moistness caused by perspiration, and the sensitivity of the sensing technology to the human skin. Moreover, we noticed that the formation of the fingerprint pattern in the image sequence shows a homogeneous pattern development which can be mainly explained by the 3-D shape of the fingertip and the fingerprint elasticity. Additionally, the spatio-temporal methods have the capacity to detect anomalous patterns caused by the various PAI species. For example, the development of the contours of the fingerprint impression for some attack species such as gelatin and latex show rough edges in the early frames of the presentation sequence, consequently, enhance the PAD subsystem’s accuracy. Based on these observation we conclude that dynamic acquisition provides more information in comparison with analysing static fingerprint images.

The local spatio-temporal features were extracted using VLBP and LBP-TOP. On the other hand, spectral features were explored locally using VLPQ and LPQ-TOP, and globally using GIST 3-D. These feature extractors are evaluated for a thermal and an optical sensors showing an advantage for the latter due to its acquisition characteristics.

The experiment points out the importance of studying each sensing technology apart through comparing (i) the accuracy of the different feature extractors, and (ii) the potential of the attack species on the two sensors. The best accuracy is obtained by LBP-TOP for the optical sensor with 1.11 BPCER20, and by LPQ-TOP for the thermal sensor with 3.89 BPCER20.

These results would seem to suggest that our approach has an excellent capability of eliminating/mitigating PAs in different sensing technologies. Further, a comparison with SoA mechanisms shows that our method provides competitive error rates. However, given the small number of participants in the database, caution must be taken.

Our results are promising and should be validated by a larger database with additional attack species and sensing technologies. We recommend that further research should concentrate on fingerprint specific dynamic features such as the variation of fingerprint quality during the presentation.

## Figures and Tables

**Table 1:** Performance analysis for the SoA fingerprint PAD mechanisms (reported in [4,5]).

PAD Category	Reference	PAI Species	Sensor/s	APCER (%)	BPCER (%)	APCER = BPCER
Distortion Analysis	Antonelli 2006 [6]	Gelatin, RTV silicon, white glue, and latex	Optical	-	-	11.24
	Zhang 2009 [7]	Silicon	Optical	-	-	4.5
	Jia 2007 [8]	Gelatin	Capacitive	-	-	4.87
Perspiration Analysis	Derakhshani 2003 [9]	Play-Doh, cadaver	Capacitive	-	-	11.11
	Parthasaradhi 2005 [10]	Play-Doh, cadaver	Capacitive	5–20	6.77–20	-
			Optical	4.6–14.3	0–26.9	
			Electro-Optical	0–19	6.9–38.5	
	Abhyankar 2009 [11]	Play-Doh, cadaver, and gummy	Capacitive	-	-	13.85
			Optical			
			Electro-Optical			
	Plesh 2019 [12]	Paper print, transparent film, wood glue, latex, Play-Doh, ecoflex, gelatin, dragonskin, ModelMagic, and SillyPutty	Optical	0.02	13.8–18.35	-
	Husseis 2020 [5]	Play-Doh, gelatin, white glue, spray rubber, nail hardener, nail polish, and latex.	Optical	5	19.5	13
			Thermal	5	18.1	9.5

**Table 2:** The used feature extraction (FE) algorithms.

FE Algorithm	FE Mode	Domain of FE	Source of Features	Reference
GIST 3-D	Mode 1	spatio-temporal frequency domain	Sub-volumes in the frequency domain	[23]
Volume Local Binary Patterns	Mode 2	spatio-temporal domain	3-D Patches	[20]
Local Binay Patterns from Three Orthogonal Planes	Mode 3	spatio-temporal domain	Patches of TOPs	[20]
Volume Local Phase Quantization	Mode 2	spatio-temporal frequency domain	3-D Patches	[25]
Local Phase Quantization from Three Orthogonal Planes	Mode 3	spatio-temporal frequency domain	Patches of TOPs	[25]

**Table 3:** Fingerprint presentation types in the database (the same protocol is applied for the 2 sensors).

Presentation Type	Visit/PAI Species	Number of Presentations Per Sensor
**Bona fide**	visit 1	198
	visit 2	198
**Attack**	Play-Doh	198
	Gelatin	198
	White glue	198
	Spray rubber	198
	Nail hardener	198
	Nail polish	198
	Latex	198
**Total**	_	**1782**

**Table 4:** Comparison between the characteristics of the sensors and presentations in the database.

Sensing Technology	Resolution	Image Size	Size of the Segmented Images	Scan Time	Number of Frames Per Presentation
Optical	500 ppi	900×900 pixels	depends on the touched surface for each presentation	0.05 s/image	Varies with respect to the user’s presentation time with average of 25 frames/presentation
Thermal	385 ppi	180×256 pixels	90×128 pixels	0.7 s/image	7

**Table 5:** PAD subsystem performance for the optical sensor.

Descriptor	at TEER			at APCER = 5%			at APCER = 2.5%		
	TEER	Successful Attacks	Rejected Bona Fide	BPCER20	Successful Attacks	Rejected Bona Fide	BPCER	Successful Attacks	Rejected Bona Fide
$VLPQ_{3,3}$	**5.56%**	35	10	**5.56%**	31	10	**8.89%**	16	16
$VLPQ_{5,5}$	6.11%	39	11	6.67%		12	11.11%		20
$VLPQ_{7,7}$	9.21%	58	17	15.00%		27	29.44%		53
$VLPQ_{9,9}$	9.44%	60	17	12.22%		22	25.00%		45
$LPQ-TOP_{3,3,3}$	5.08%	32	9	5.56%	31	10	9.44%	16	17
$LPQ-TOP_{5,5,5}$	**3.89%**	25	7	**3.89%**		7	**7.22%**		13
$LPQ-TOP_{7,7,7}$	6.11%	39	11	6.67%		12	7.78%		14
$LPQ-TOP_{9,9,9}$	5.56%	35	10	6.11%		11	11.11%		20
			0						
GIST 3-D	**5.56%**	35	10	**6.67%**	31	12	**9.44%**	16	17
$VLBP_{1,4,1}$	3.65%	23	7	1.67%	31	3	7.22%	16	13
$VLBP_{1,4,3}$	4.76%	30	9	4.44%		8	11.67%		21
$VLBP_{2,4,1}$	**2.78%**	18	5	**1.67%**		3	**5.00%**		9
$VLBP_{2,4,3}$	3.65%	23	7	2.22%		4	5.56%		10
$VLBP_{1,4,1}^{ri}$	5.00%	32	9	5.00%	31	9	8.89%	16	16
$VLBP_{1,4,3}^{ri}$	6.67%	42	12	8.33%		15	13.33%		24
$VLBP_{2,4,1}^{ri}$	3.89%	25	7	3.33%		6	5.00%		9
$VLBP_{2,4,3}^{ri}$	4.92%	31	9	4.44%		8	8.89%		16
$LBP-TOP_{1,8,1}$	4.44%	28	8	3.89%	31	7	5.00%	16	9
$LBP-TOP_{1,8,3}$	3.97%	25	7	2.22%		4	5.56%		10
$LBP-TOP_{2,8,1}$	3.65%	23	7	2.78%		5	3.89%		7
$LBP-TOP_{2,8,3}$	3.89%	25	7	2.78%		5	3.89%		7
$LBP-TOP_{1,8,1}^{u2}$	4.76%	30	9	4.44%	31	8	7.22%	16	13
$LBP-TOP_{1,8,3}^{u2}$	**2.22%**	14	4	**1.11%**		2	**2.22%**		4
$LBP-TOP_{2,8,1}^{u2}$	5.56%	35	10	5.56%		10	7.22%		13
$LBP-TOP_{2,8,3}^{u2}$	3.49%	22	6	2.22%		4	6.11%		11

**Table 6:** PAD subsystem performance for the thermal sensor.

Descriptor	at TEER			at APCER = 5%			at APCER =2.5%		
	TEER	Successful Attacks	Rejected Bona Fide	BPCER20	Successful Attacks	Rejected Bona Fide	BPCER	Successful Attacks	Rejected Bona Fide
$VLPQ_{3,3}$	**8.10%**	51	15	**13.89%**	31	25	**27.78%**	16	50
$VLPQ_{5,5}$	13.02%	82	23	31.67%		57	46.11%		83
$VLPQ_{7,7}$	17.94%	113	32	46.67%		84	65.00%		117
$LPQ-TOP_{3,3,3}$	**4.92%**	31	9	**3.89%**	31	7	**14.44%**	16	26
$LPQ-TOP_{5,5,5}$	7.30%	46	13	9.44%		17	17.78%		32
$LPQ-TOP_{7,7,7}$	6.67%	42	12	8.89%		16	22.78%		41
GIST 3-D	**12.22%**	77	22	**28.89%**	31	52	**46.67%**	16	84
$VLBP_{1,4,1}$	**12.86%**	81	23	**30.00%**	31	54	**51.67%**	16	93
$VLBP_{1,4,3}$	16.19%	102	29	27.22%		49	48.33%		87
$VLBP_{2,4,1}$	16.03%	101	29	37.22%		67	61.11%		110
$VLBP_{2,4,3}$	19.44%	123	35	43.89%		79	57.78%		104
$VLBP_{1,4,1}^{ri}$	12.78%	81	23	23.89%	31	43	41.11%	16	74
$VLBP_{1,4,3}^{ri}$	12.70%	80	23	37.22%		67	72.22%		130
$VLBP_{2,4,1}^{ri}$	16.19%	102	29	33.33%		60	53.89%		97
$VLBP_{2,4,3}^{ri}$	13.33%	84	24	35.56%		64	56.67%		102
$LBP-TOP_{1,8,1}$	7.78%	49	14	10.56%	31	19	23.33%	16	42
$LBP-TOP_{1,8,3}$	8.33%	53	15	16.67%		30	24.44%		44
$LBP-TOP_{2,8,1}$	7.46%	47	13	10.00%		18	20.00%		36
$LBP-TOP_{2,8,3}$	**7.22%**	46	13	**15.00%**		27	**36.11%**		65
$LBP-TOP_{1,8,1}^{u2}$	7.78%	49	14	14.44%	31	26	28.33%	16	51
$LBP-TOP_{1,8,3}^{u2}$	8.33%	53	15	13.33%		24	28.33%		51
$LBP-TOP_{2,8,1}^{u2}$	8.33%	53	15	12.78%		23	29.44%		53
$LBP-TOP_{2,8,3}^{u2}$	8.25%	52	15	15.00%		27	37.22%		67

**Table 7:** BPCER20 comparison between the optical and thermal sensors.

Sensor\FE	VLPQ		LPQ-TOP		GIST 3-D	VLBP		LBP-TOP	
**Optical**	**$VLPQ_{3,3}$**	5.56%	**$LPQ-TOP_{5,5,5}$**	3.89%	6.67%	$VLBP_{2,4,1}$	1.67%	$LBP-TOP_{1,8,3}^{u2}$	2.22%
**Thermal**	**$VLPQ_{3,3}$**	13.89%	**$LPQ-TOP_{3,3,3}$**	3.89%	28.89%	$VLBP_{1,4,1}$	30.00%	$LBP-TOP_{2,8,3}$	15.00%
**Difference**	8.33%		**0.00%**		22.22%	28.33%		12.78%	

**Table 8:** Attacks strength considering different PAI species (optical sensors).

Feature Extractor	SVM Error Rates		APCER_PAI_						
	APCER	BPCER	PlayDoh	White Glue	Spray Rubber	Polish Nail	Nails Hardener	Gelatin	Latex
**$VLBP_{2,4,1}$**	1.75%	7.78%	**0.00%**	1.11%	1.11%	**0.00%**	8.89%	**0.00%**	1.11%
**LBP−TOP1,8,3**	1.59%	6.67%	3.33%	1.11%	**0.00%**	1.11%	1.11%	2.22%	2.22%
**$VLPQ_{3,3}$**	3.33%	6.67%	5.56%	**0.00%**	3.33%	1.11%	8.89%	4.44%	**0.00%**
**$LPQ-TOP_{5,5,5}$**	2.38%	11.67%	3.33%	3.33%	**0.00%**	**0.00%**	3.33%	4.44%	2.22%
**GIST 3−D**	1.43%	10.56%	4.44%	1.11%	2.22%	1.11%	**0.00%**	1.11%	**0.00%**

**Table 9:** Attacks strength considering different PAI species (thermal sensors).

Feature Extractor	SVM Error Rates		APCER_PAI_						
	APCER	BPCER	PlayDoh	White Glue	Spray Rubber	Polish Nail	Nails Hardener	Gelatin	Latex
**$VLBP_{1,4,1}$**	1.59%	56.11%	**0.00%**	10.00%	1.11%	**0.00%**	**0.00%**	**0.00%**	**0.00%**
**LBP−TOP2,8,3**	4.44%	16.67%	1.11%	21.11%	6.67%	2.22%	**0.00%**	**0.00%**	**0.00%**
**$VLPQ_{3,3}$**	3.33%	18.33%	2.22%	15.56%	1.11%	1.11%	**0.00%**	3.33%	**0.00%**
**$LPQ-TOP_{3,3,3}$**	2.70%	11.11%	**0.00%**	8.89%	4.44%	2.22%	**0.00%**	3.33%	**0.00%**
**GIST 3−D**	4.76%	29.44%	8.89%	24.44%	**0.00%**	**0.00%**	**0.00%**	**0.00%**	**0.00%**

**Table 10:** Performance comparison with the SoA dynamic PAD mechanisms.

Sensing Tech.	PAD Mechanism	APCER (%)	BPCER (%)	TEER (%)
Optical	Antonelli	-	-	11.24
	Zhang	-	-	4.5
	Parthasaradhi	4.6–14.3	0–26.9	-
	Abhyankar	-	-	13.85
	Plesh	0.02	13.8–18.35	-
	Husseis	5	19.5	13
	**Proposed**	**5**	**1.11**	**2.22**
Thermal	Husseis	5	18.1	19.5
	**Proposed**	**5**	**3.89**	**4.92**

## Data Availability

Not applicable.
